# Case Report: Innovative radiotherapy strategies in advanced colon cancer with oligometastasis: a case of online adaptive radiotherapy and lattice stereotactic re-irradiation

**DOI:** 10.3389/fonc.2026.1713282

**Published:** 2026-03-12

**Authors:** Ying Li, Xing Luo, Ningyue Xu, Jiatong Zeng, Jie Zhang, Tao Ren, Fei Cao

**Affiliations:** 1 School of Clinical Medicine, Chengdu Medical College, Chengdu, China; 2 Oncology Department, The First Affiliated Hospital of Chengdu Medical College, Chengdu, China

**Keywords:** ascending colon cancer, lung metastasis, online adaptive radiotherapy (oART), re-irradiation, spatially fractionated radiotherapy (SFRT)

## Abstract

Conventional radiotherapy demonstrates limited efficacy in advanced colon cancer with bulky radio-resistant metastatic lesions. A case report detailing the outcomes and toxicity profile of a 71-year-old female with progressive colon cancer and pulmonary oligometastasis treated using an individualized radiotherapy approach is presented. The patient, with an ECOG performance status of 3, presented severe symptoms including partial intestinal obstruction, intractable cough, and hemoptysis following multiple lines of systemic therapy and prior intensity-modulated radiotherapy (IMRT) to lung metastases (5750 cGy in 23 fractions, delivered 17months earlier). Following multidisciplinary evaluation, online adaptive radiotherapy (oART) using a fan-beam CT-guided linear accelerator system was administered to the ascending colon primary tumor (50 Gy in 25 fractions). Concurrently, spatially fractionated radiotherapy (SFRT) was employed for the radio-resistant bulky pulmonary lesion: lattice radiation therapy (LRT) PTVpeak (22.5 Gy in 3 fractions) followed by sequential conventional fractionation to PTV (30 Gy in 15 fractions). No acute radiotherapy-related adverse events were observed. At 11-month follow-up, both target lesions exhibited partial response (PR) on imaging, accompanied by complete alleviation of intestinal obstruction and hemoptysis. Consequently, the patient’s performance status improved to ECOG 1. oART appears to be a viable approach for symptom palliation and disease control in advanced colon cancer with emergent complications, particularly among patients with compromised functional status. Additionally, SFRT shows potential efficacy in re-irradiation settings for bulky, radio-resistant lesions. The combined use of adaptive precision radiotherapy and spatially fractionated techniques may therefore represent an emerging therapeutic paradigm for oligometastatic colon cancer, meriting further evaluation in prospective clinical trials.

## Introduction

Colorectal cancer (CRC) is the third most prevalent malignancy worldwide and ranks as the second leading cause of cancer-associated mortality (1). In advanced disease stages, 30-50% of patients undergo pulmonary metastases, underscoring the critical role of chemoradiotherapy in multidisciplinary management (2, 3). Nevertheless, pelvic radiotherapy delivery remains technically challenging due to interfractional organ motion, anatomical variations in rectal distension, colonic content and bladder filling induce significant target displacement (4–7), while colonic peristalsis and the inherent mobility of unfixed mesenteric structures further compromise targeting precision. These dynamic complexities persist even with advanced techniques like intensity-modulated radiotherapy (IMRT) and volumetric modulated arc therapy (VMAT), leading to suboptimal dose conformity and increased risks of normal tissue toxicity (8).

Emerging oART systems address these limitations through real-time anatomical feedback and dynamic plan re-optimization (9, 10). Unlike conventional image-guided radiotherapy (IGRT) that relies on static planning CT datasets, oART establishes a closed-loop workflow capable of accounting for interfractional organ deformation and tumor volume changes. This approach enables margin reduction while maintaining target coverage by adaptively redefining the planning target volume (PTV) during treatment delivery, thereby minimizing irradiated normal tissue volumes and associated toxicity risks (11, 12).

Moreover, spatially fractionated radiotherapy (SFRT) has re-emerged as a promising strategy for bulky radio-resistant tumors (13–16). Modern implementations utilizing grid – based VMAT create steep dose gradients that selectively spare critical structures while delivering ablative doses to aggressive tumor sub-volumes (17). This technique not only overcomes conventional dose limitations for large lesions but may also potentiate systemic antitumor immunity through immunomodulatory effects (17–20).

This work reports on a patient with refractory ascending colon cancer and lung metastases who progressed after multiple lines of chemotherapy and radiotherapy to the lung lesion and subsequently presented with intestinal obstruction and hemoptysis. Following multidisciplinary evaluation, re-treatment with a combination of oART and SFRT was undertaken as a practical application of this cutting edge therapeutic approach (21, 22).

## Case presentation

**Figure 1:**
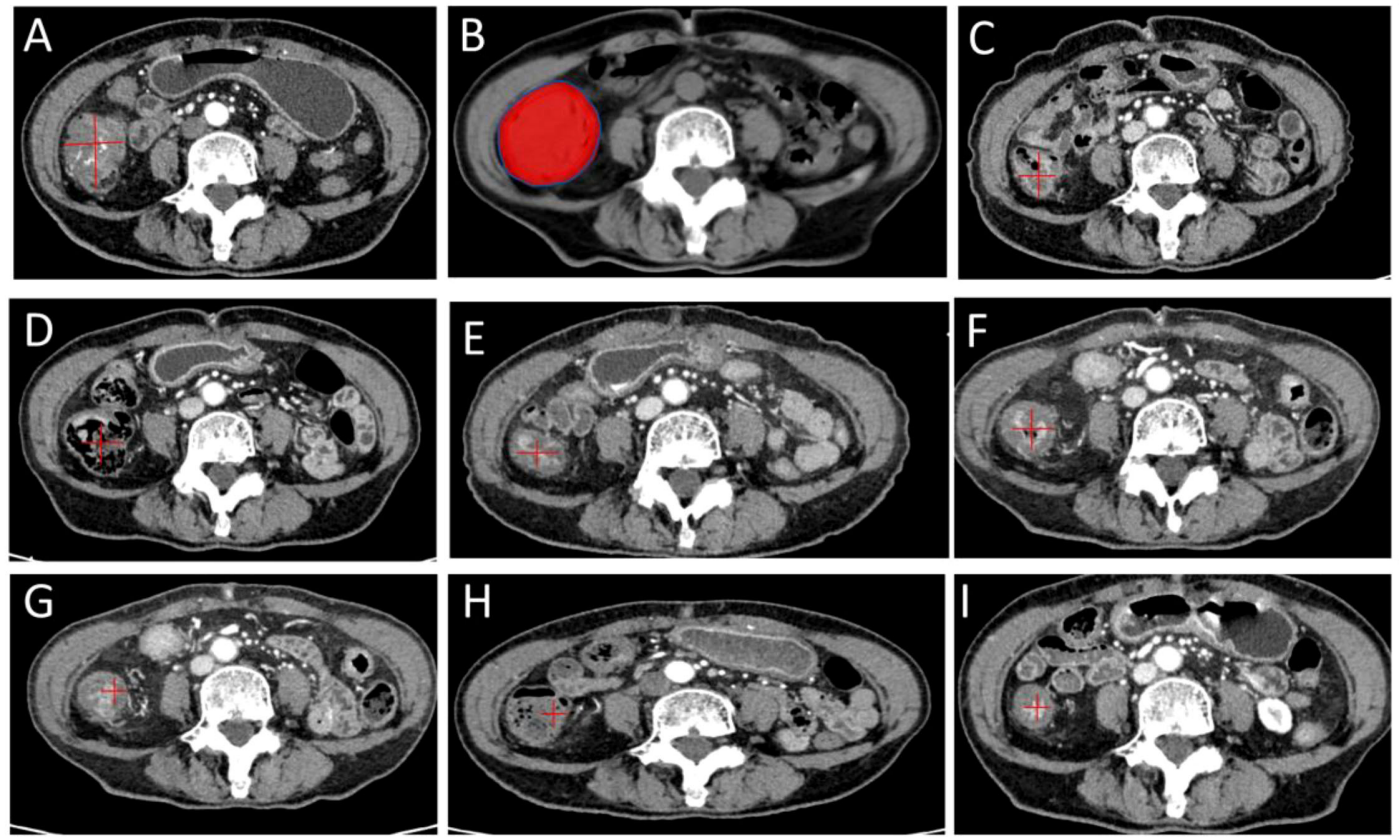
Serial CT imaging (1mm) of an ascending colon cancer lesion during oART: Baseline contrast-enhanced CT before treatment **(A)**; Online adaptive radiotherapy (ART) dose distribution, showing the 100% prescription isodose (red) providing conformal coverage of the planning target volume **(B)**; Follow-up CT scans obtained at various time points (26, 75, 120, 200, 230, 263 and 330 days post-radiotherapy) demonstrating continuous tumor regression **(C–I)**.

**Figure 2:**
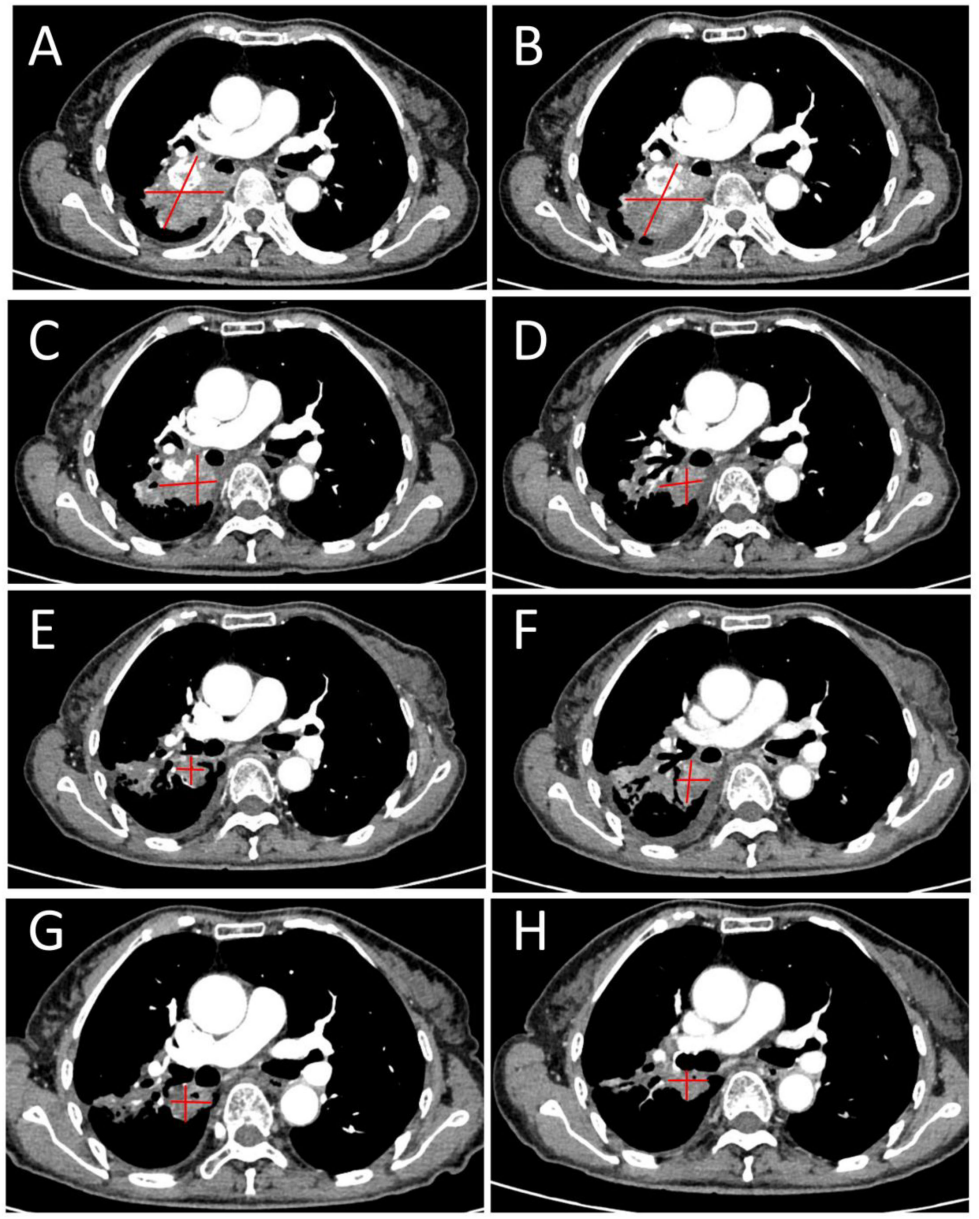
Serial evolution of a right lung metastasis treated with SFRT: Baseline pre-treatment imaging **(A)**; Baseline pre-SFRT scan (obtained 26 days after completion of colorectal radiotherapy) **(B)**; Follow-up scans at various time points post-SFRT (26, 75, 120, 200, 266, and 288 days) **(C-H)**. The right lung metastasis (indicated by red arrows) demonstrates gradual regression and reduction of solid components.

A 71-year-old female diagnosed with ascending colon adenocarcinoma (Stage IV, T3NxM1) carrying KRAS c.34G>T (p.G12C) mutation and demonstrating microsatellite stability (MSS) at Sichuan Provincial People’s Hospital on August 11, 2022. At presentation, the patient had an Eastern Cooperative Oncology Group (ECOG) performance status of 3 and a Numerical Rating Scale (NRS) pain score of 5. Clinical presentation included bulky pulmonary oligometastases as the primary metastatic manifestation. The treatment chronology is as follows: First-line therapy with 7 cycles of capecitabine/oxaliplatin (CapeOX) chemotherapy and intensity-modulated radiation therapy (IMRT: 5750 cGy) delivered in 23 fractions targeting pulmonary oligometastases,the Lung_all V20 = 27.42%, Lung_R V20 = 47.84%,.The second-line therapy was with FOLFIRI regimen (leucovorin, fluorouracil, irinotecan) in combination with bevacizumab administered in 3 cycles following radiographic confirmation of disease progression. Then third-line therapy was using raltitrexed plus regorafenib combination therapy (2cy). By July 2024, the patient developed acute complications including mechanical bowel obstruction (manifesting as abdominal pain and melena) along with progressive pulmonary metastatic symptoms (hemoptysis and intractable cough). There was a local progression of the primary colonic lesion and interval increase in pulmonary metastatic burden, supporting clinical deterioration (See **Figures 1**, **2**).

**Figure 3:**
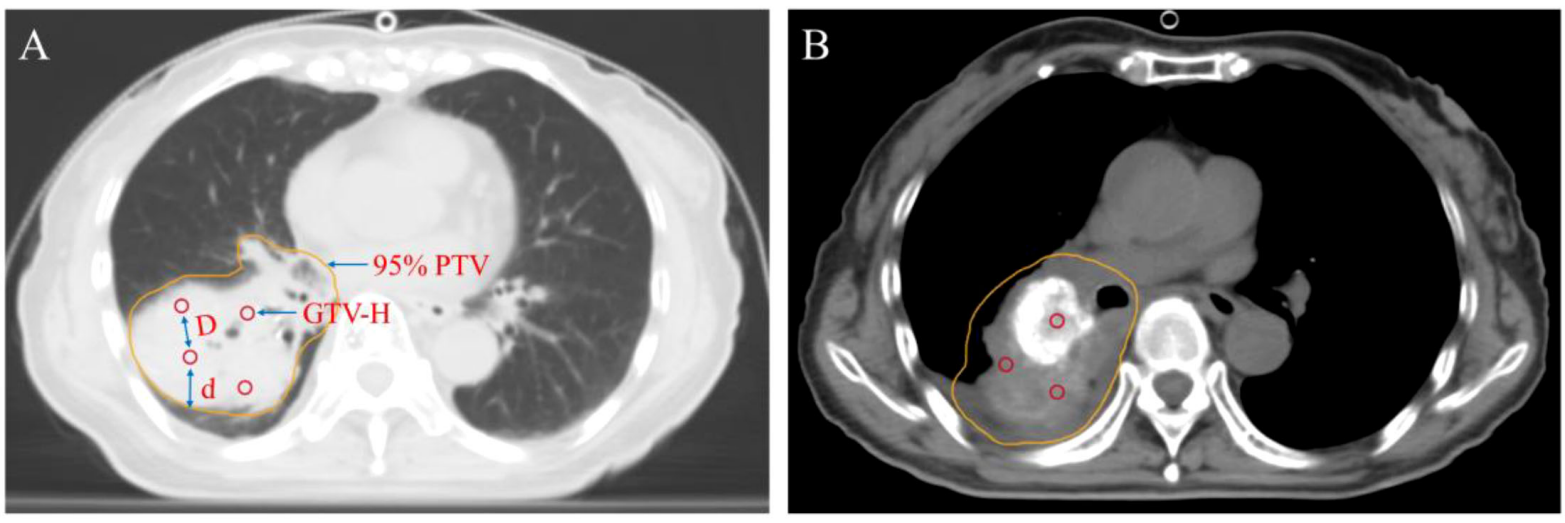
Target volume design for pulmonary lesions in SFRT: Lung window **(A)**; Mediastinal window **(B)**. GTV-H: For a 1.0 cm diameter sphere, d: represents the distance between GTV-H and the 95% PTV margin, ranging from 1.5 to 2.5 cm; D: denotes the distance between GTV-H, ranging from 2.0 to 3.0 cm. 95% PTV: 95% PTV receiving the prescribed dose.

**Figure 4:**
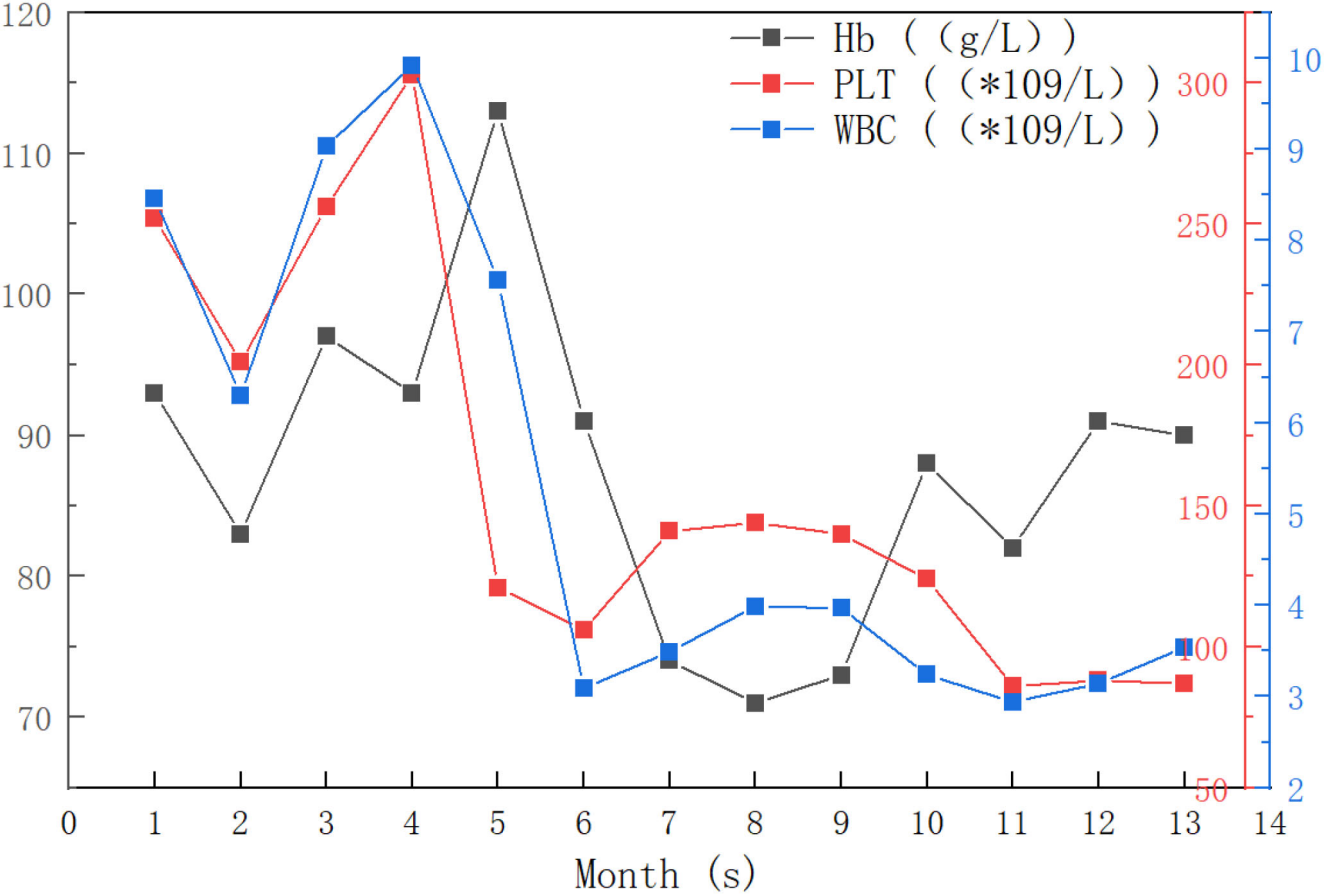
Longitudinal monitoring of hematological parameters during 13 months of sequential therapy with online adaptive radiotherapy (ART) and spatially fractionated radiotherapy (SFRT): No grade ≥3 hematological toxicity occurred.

A customized radiation approach was implemented to address the limitations of conventional radiotherapy in colonic malignancies, particularly suboptimal target reproducibility and dose conformity, combined with the therapeutic challenges posed by radio-resistant pulmonary oligometastases in a patient with prior thoracic IMRT (57.5 Gy/23 fractions) 17 months ago (cumulative spinal cord dose: 3300 cGy). For the primary colonic tumor, online adaptive radiotherapy (oART) was administered in 25 fractions (total dosage 50 Gy) at 5 fractions per week, utilizing real-time deformable image registration to account for intestinal motility, with dual objectives of obstruction relief and pain management. For re-irradiation of pulmonary oligometastases using SFRT, the following spatial segmentation geometry were implemented to achieve dose constraints for normal organs according to RTOG guidelines while minimizing overlap with previous conventional fields (23, 24). First, we rigidly registered the patient’s prior radiotherapy plan with current CT images to precisely delineate historical high-dose regions (107% of prescribed dose). In designing the grid, high-dose peaks were intentionally placed in historically low-dose or unirradiated regions. Each peak was a 1.0 cm diameter sphere spaced 2.0 – 3.0 cm apart. Peaks maintained a 1.5 – 2.5 cm margin from PTV edges and critical organ-at-risk (OAR) borders. A single dose of 7.5 Gy was delivered over 3 fractions. Placement avoided spinal cord, esophagus, major vessels, heart, brachial plexus, trachea and muscular-fascial structures while ensuring valley regions covered historical high-dose zones (25). Dose-volume histogram verification confirmed cumulative doses remained within safety margins (**Figure 3**). This sequential regimen established a 3.75:1 dose gradient between PTV peak and PTV while maintaining strict organ-at-risk constraints (spinal cord max dose < 8 Gy). Twice-weekly biochemical surveillance (complete blood count, renal/hepatic function, electrolytes) demonstrated parameters consistently within normal limits throughout treatment, with no CTCAE v5.0-graded toxicities observed. Subsequent therapy with TAS-102 plus bevacizumab achieved sustained clinical benefit: ECOG performance status 1, complete resolution of pain (NRS 0), restoration of normal bowel function, and cessation of hemoptysis with cough improvement significantly. At 11-month follow-up, durable treatment response without radiation-related adverse events was confirmed. Contrast-enhanced CT evaluation per RECIST 1.1 criteria demonstrated partial response (PR) in both target lesions(See **Tables 1**, **2**; **Figure 4**).

**Table 1:** Treatment response and clinical symptoms of intestinal tumor.

Parameter	Baseline	Day 26	Day 75	Day 120	Day 200	Day 230	Day 263	Day 330
Largest Diameter (mm)	43.79	26.55	10.69	20.11	17.22	18.16	17.16	16.10
Change Rate (%)	—	-39.37	-75.59	-54.08	-60.68	-58.53	-60.81	-63.23
Abdominal Pain (NRS 0-10)	5/10	3/10	1/10	1/10	0/10	0/10	0/10	0/10
Bowel Obstruction (%)	90%	75%	45%	30%	—	—	—	—
Toxicity (Grade)	—	0	1	1	1	0	0	0
ECOG Score	3	3	1	1	1	1	1	1

1. Radiotherapy Regimen: The intestinal tumor was treated with Online Adaptive Radiotherapy (oART) guided by FBCT, with a total dose of 50 Gy in 25 fractions.

2. Change Rate Calculation: The change rate was calculated relative to the Baseline data.

3. Symptoms and Assessment: Abdominal pain was assessed using the Numerical Rating Scale (NRS, 0-10); bowel obstruction is presented as a percentage of luminal narrowing, with “—” indicating no obstruction.

4. Toxicity: Graded according to the Common Terminology Criteria for Adverse Events (CTCAE) (Grade 0: None, Grade 1: Mild).

5. ECOG Score: Eastern Cooperative Oncology Group performance status score (range 0-5, with higher scores indicating greater disability).

**Table 2:** Treatment response and clinical symptoms of lung tumor.

Parameter	Baseline	Day 26	Day 75	Day 120	Day 200	Day 266	Day 288
Largest Diameter (mm)	53.19	39.85	29.64	22.66	22.47	21.16	30.28
Change Rate (%)	—	-25.15	-44.33	-57.44	-57.80	-60.26	-43.13
Cough	+++	+++	++	+	±	±	±
Hemoptysis	+++	+++	++	—	—	—	—
ECOG Score	3	3	1	1	1	1	1

1. Radiotherapy Regimen: The lung tumor was treated with SFRT. The dose was prescribed as follows: GTV-H: 22.5 Gy/3Fx; 95% PTV: 30 Gy/15Fx.

2. Change Rate Calculation: The change rate was calculated relative to the Baseline data.

3. Symptom Score: Cough and hemoptysis were assessed using a qualitative scale: “+++” indicates severe, “++” indicates moderate, “+” indicates mild, “±” indicates minimal, and “—” indicates absent.

### ART in colon cancer

Traditional radiotherapy planning relies on an initial 3D data set from CT simulation, where normal tissues and tumor contours are delineated to generate optimized radiotherapy planning. Once the plan is finalized, the patient receives a fixed radiation dose over multiple treatment sessions, typically once daily. Adaptive Radiotherapy (ART), as a feedback-driven technology, can monitor anatomical changes and tumor dynamics in real-time during treatment, dynamically adjusting the treatment plan accordingly (26). In comparison to traditional radiotherapy, ART significantly improves target coverage while reducing toxicity. Consequently, maximizing the tumor dose while ensuring normal tissue doses remain within acceptable limits (27) (See **Tables 3**, **4**).

**Table 3:** Comparison of ART and IGRT plans in terms of target volume dosimetry (P25, P75).

Group	ARTplan(n=24)	IGRTplan(n=24)	Z	P
D_max_	5273.85(5269.58,5276.21)	5436.05(5388.49,5792.69)	-5.897	0.000
D_98_	4987.36(4985.75,5002.44)	103.24(68.34,201.9)	-5.938	0.000
D_95_	5016.86(5015.27,5025.85)	140.75(88.83,268.12)	-5.938	0.000
D_50_	5122.04(5117.12,5127.66)	4817.58(4562.62,5014.8)	-5.691	0.000
D_2_	5230.07(5226.29,5233.41)	5290.72(5230.39,5303.61)	-3.114	0.002
HI	0.0468(0.0455,0.0476)	1.0721(1.0168,1.1489)	-5.938	0.000
CI	0.9430(0.9356,0.9464)	0.3437(0.2745,0.3880)	-5.938	0.000
V_100%_	97.0000(97.0000,98.1400)	43.5550(37.7150,51.2200)	-6.051	0.000

P25 and P75 represent the 25th and 75th percentiles of the dose parameters, respectively.

D_max_, Maximum dose; D_98_, D_95_, D_50_, D_2_, Doses received by 98%, 95%, 50%, and 2% of the PTV volume, respectively; CI, Conformity Index= (V_t,ref_/V_t_) · (V_t,ref_/V_ref_), where V_t,ref_ is the target volume covered by the reference isodose line, V_t_ is the target volume, and V_ref_ is the total volume covered by the reference isodose line; HI, Homogeneity Index = (D_2_ - D_98_)/D_50_; V_100%_, Volume percentage of PTV covered by 100% of the prescription dose.

**Table 4:** Dosimetric comparison of organs at risk between ARTplan and IGRTplan (P25, P75).

Group	ARTplan(n=24)	IGRTplan(n=24)	Z	P
D_max_	4335.42(4303.15,4416.56)	5726.46(5449.75,5843.82)	-5.92	0.000
V_30_	2.14 (1.83,2.60)	2.24 (1.84,2.98)	-0.85	0.398
D_mean_	1044.15 (905.88,1305.36)	152.66 (79.38,355.64)	-5.82	0.000

D_max_, Maximum dose; V_30_, Volume percentage receiving 30Gy dose (covering the corresponding OAR); D_mean_, Mean radiation dose to the right kidney (Kidney-R). Organs-at-Risk (OARs) include the bowel segment (Bowel Bag_) and the right kidney (Kidney-R).

ART is categorized into three based on the rate and nature of anatomical changes (27), namely offline, online and real time ART. With offline ART, plan adjustments are made between treatment sessions and it is suitable for slow anatomical changes such as loss of weight. Plans are re-optimized immediately before each treatment with online ART, and it is suitable for rapid anatomical changes​ such as bladder or rectal filling or organ motion. Real-time ART utilizes real-time motion monitoring and/or gating, such as respiratory motion compensation. The optimal timing, frequency and strategy for each ART type depend on the potential clinical benefits, local anatomical changes and clinical context. Online ART, for instance, addresses unpredictable anatomical changes during treatment and the positional changes of abdominal organs caused by peristalsis such as cervical complex motion, bladder filling and colonic peristalsis. This requires the ability to acquire in-time target images to adjust the treatment plan accordingly. Therefore, online ART necessitates daily high-quality imaging, rapid re-contouring, re-planning, and re-evaluation to ensure accuracy and safety. Efficient workflow and clinical resource allocation​ are therefore, very critical (27–29).

Current online ART relies on cone-beam/fan-beam CT (CBCT/FBCT) or hybrid MRI-guided linear accelerator systems. Although CT and MRI are not entirely equivalent in imaging segmentation, the scanning images must ensure overall dosimetric uncertainty within ±5% and spatial uncertainty within ±5 mm (30, 31).

In this case, online ART guided by uRT-Linac 506c KV-FBCT (Shanghai Lianying, Shanghai, China) was used to treat the ascending colon lesion, achieving excellent target conformity. FBCT is extensively utilized in IGRT due to its minimal infrastructure requirements, reduced training demands, short image acquisition time (2–3 minutes) and broad clinical applicability, including suitability for patients with metal implants, larger body size, or claustrophobia (32, 33). Numerous studies have demonstrated the successful application of FBCT-guided online ART in abdominal and pelvic tumors (34) and cervical cancer (9, 35, 36) with median re-planning time from FBCT acquisition to treatment initiation being not more than 20 minutes.

Although ART workflows are largely automated within integrated platforms, FBCT-guided online ART still requires manual verification and refinement of OAR and tumor contours by radiation oncologists and medical physicists, underscoring the critical role of expert human intervention in ensuring treatment accuracy. There is an urgent need for large-scale clinical trials to define the unique advantages of ART in treating cancers at different sites. Compared to conventional radiotherapy, ART offers superior precision, however, it is time-consuming and resource-demanding, as it requires continuous monitoring (37–39).

In inoperable colon cancer treatment, selecting the optimal ART dose is highly crucial (40) since there is currently no standard for the optimal ART dose. Some studies suggest increasing the dose from 45.0 – 50.4 Gy/25 – 28Fx to 55 Gy or 60 Gy using IGRT to improve pathological complete response (pCR) rates. However, this approach has not been widely adopted as future clinical trials are needed to validate the optimal dose for colon cancer (41–43).

### Application of ART in advanced colon cancer treatment with emergency presentations

**Figure 5:**
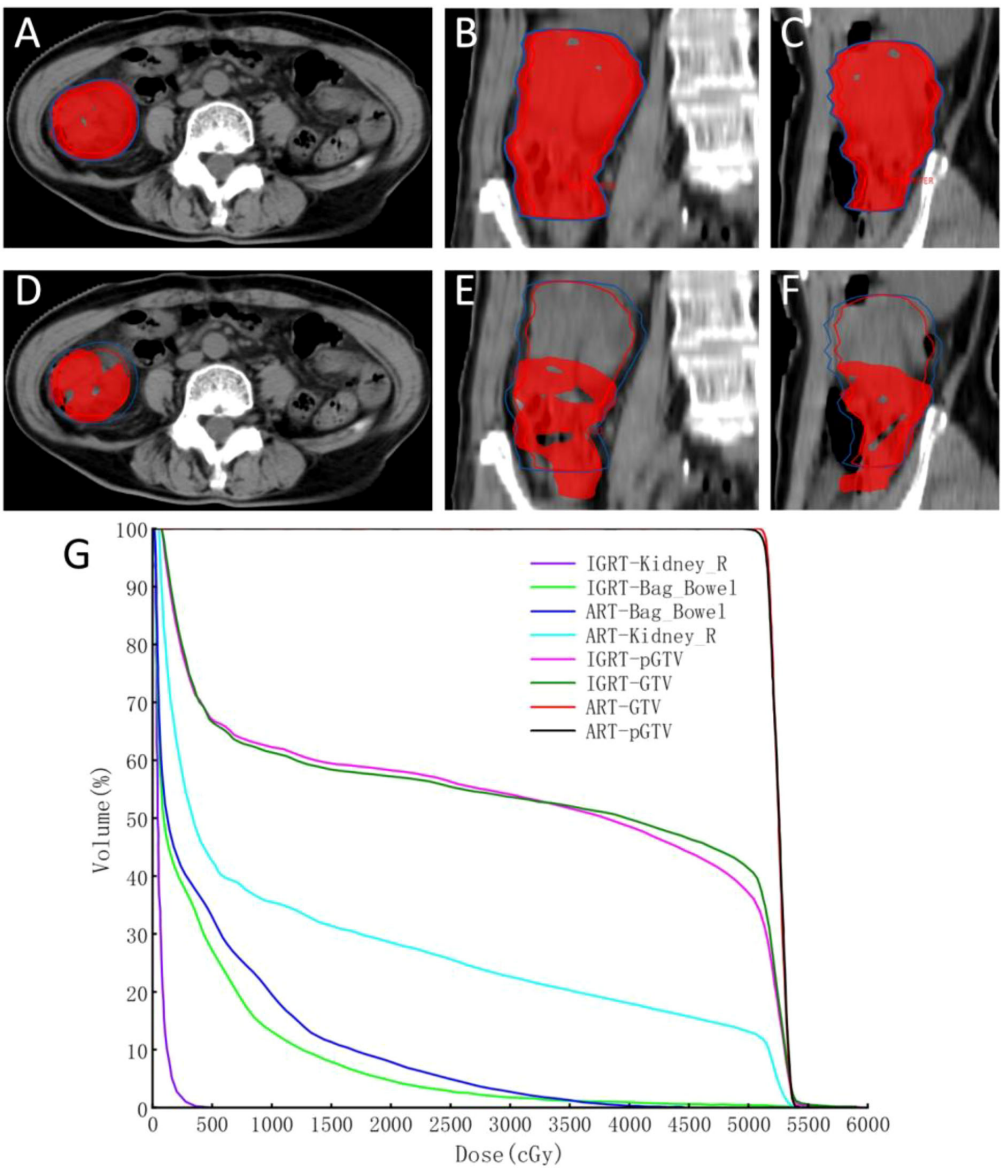
Comparison of 100% prescription isodose coverage showing the superiority of ART over IGRT: ART plan dose distribution in axial **(A)**, sagittal **(B)**, and coronal **(C)**. IGRT plan dose distribution in the same views **(D-F)**, Dose-volume histogram (DVH) comparison **(G)** ART plan (solid lines) versus IGRT plan (dashed lines).

In accordance with NCCN guidelines, the gross tumor volume (GTV) in this case was prescribed a dose of 45 – 50.4 Gy over 25 fractions, administered five times a week, with femoral head V_20_ < 50%, bladder V_50_ < 50% and small bowel V_35_ ≤ 180 cc (44). Prior to treatment, the patient preparation followed the CT planning protocol, which included fasting for at least one hour before the scan, bladder and rectal emptying, and ingestion of 500–1000 mL of water approximately 30 minutes before CT imaging. No abdominal compression device was used to avoid additional discomfort. The GTV was delineated on axial CT slices based on the visible colon tumor. During online ART, the integrated CT-Linac system utilized OAR-guided deformable registration algorithms to generate daily GTV by merging planning CT and CBCT images. The radiation oncologist manually edited the planning GTV (pGTV), extending from the top to the bottom of the radiopaque markers to ensure complete coverage of the colon tumor. Three key technical issues associated with online ART were addressed: (1) inter-fractional dose variation, arising from anatomical variations between treatment fractions; (2) intra-fractional dose variation, resulting from organ motion or changes in organ filling during a single fraction; (3) limited tumor visibility in IGRT, where tumors may not be clearly visible on CBCT or FBCT due to imaging limitations. It is worth noting that inter-fractional dose variation can be particularly significant in colon cancer, with reported maximum variations of up to 35 mm (45). However, the on-board treatment planning system of the integrated CT-Linac system compensates for these variations in real-time (46, 47). The system automatically delineates OARs daily and uses OAR-guided target volume deformation propagation to rapidly re-plan the daily treatment (average time: 32 minutes). This approach significantly reduces inter-fractional dose variation and setup uncertainty, enhancing treatment precision. Normal tissue doses in this case were within the maximum tolerated limits as reported by Bisello et al (48), confirming the safety of the treatment plan (**Figure 5**). Furthermore, the ascending colon lesion was assessed as a partial response (PR) according to RECIST 1.1 criteria after 330 days of ART treatment (see **Table 1**).

### Application of SFRT with bulky radio-resistant lung oligometastasis

SFRT achieves high clinical response rates and minimal toxicity in large-volume primary or metastatic malignancies by creating a highly heterogeneous dose distribution within the tumor (49). Compared to traditional radiotherapy, SFRT offers flexible dose distribution, high single doses, low marginal doses, high target precision, and reduced damage to adjacent organs. Research and clinical application of SFRT are advancing rapidly, demonstrating excellent local control rates in large-volume head and neck tumors (17), soft tissue sarcomas (50), lung cancer (51) and cervical cancer (52), with overall local control rates of 80% to 90% for advanced large tumors.

**Figure 6:**
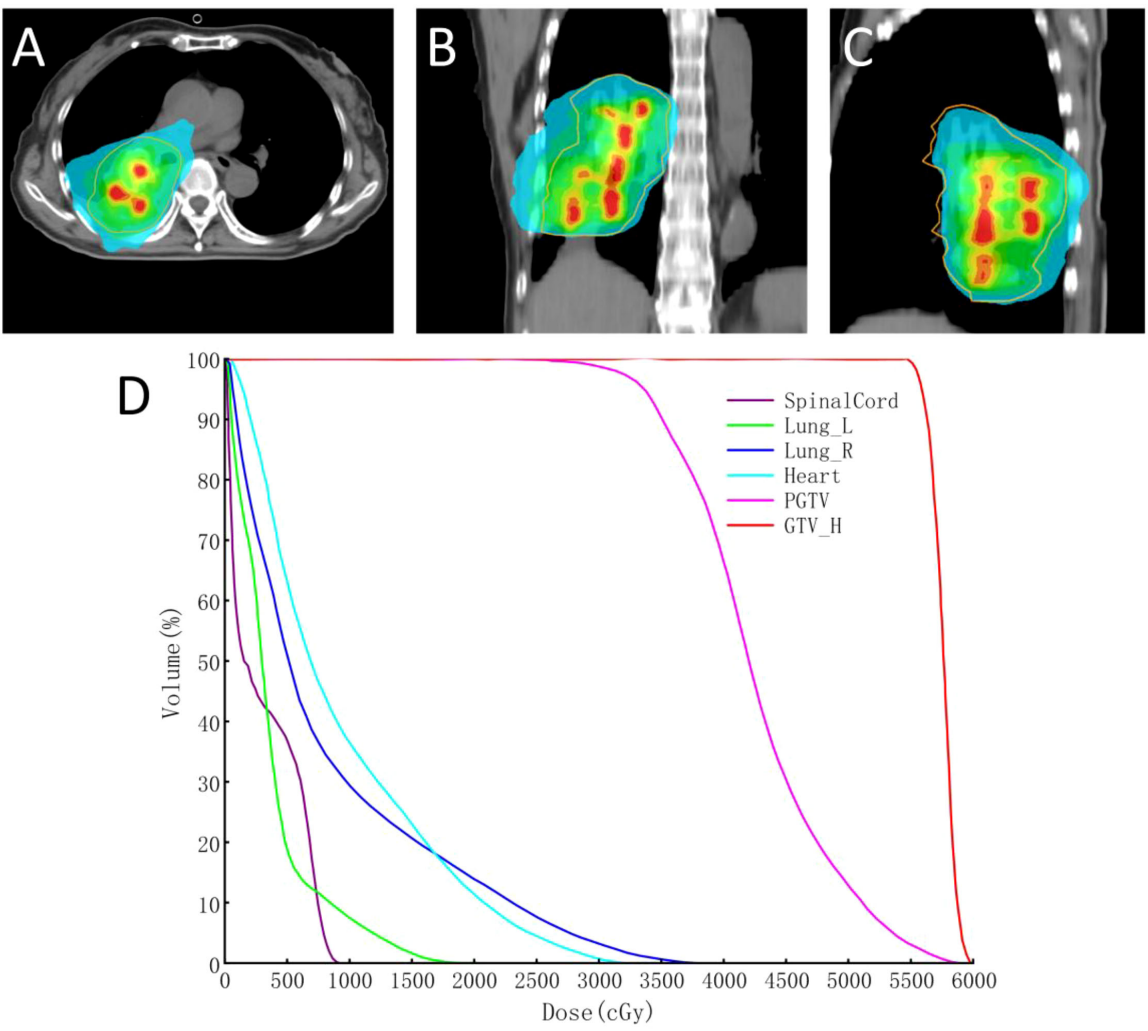
Dosimetric distribution of SFRT for the lung metastasis: Dose distribution visualization of the SFRT plan for the lung metastasis in three orthogonal views, red contour: gross tumor volume (GTV-H); green contour: 100% prescription isodose line **(A-C)**, Dose-volume histogram (DVH) of the SFRT plan **(D)**, displaying the dose-volume relationships for the GTV-H, planning target volume (pGTV), and organs at risk (heart, bilateral lungs, spinal cord).

Lattice Radiotherapy (LRT) is a novel SFRT technique (17, 53, 54). It delivers high radiation doses precisely to multiple discrete points (lattices) within the tumor. This method creates a unique dose distribution characterized by “peaks” within the tumor and “valleys” in surrounding areas, thereby enhancing tumor cell kill while minimizing exposure to normal tissues (See **Figure 3**). In comparison to conventional radiotherapy, LRT provides distinct advantages. Firstly, it reduces damage to surrounding normal tissues by confining high doses to the tumor site, offering better normal tissue protection. Secondly, it leverages bystander effects and immune activation to boost treatment efficacy and improve tumor control. Thirdly, it is particularly suitable for the treatment of large-volume or complex tumors, as well as re-irradiation cases that present challenges for conventional radiotherapy approaches (See **Figure 6**).

Considering the previous radiotherapy plan and heterogeneous biological activity of the tumor observed on contrast-enhanced CT, treatment planning was performed in accordance with RTOG guidelines for re-irradiation dosimetry with dose constraints applied to both normal organs and target volumes (EQD2,α/β=3) (24, 25, 55, 56). The organ at risk (OAR) dose constraints for the SFRT plan were as follows: right lung-PTV D_mean_ ≤ 14–16 Gy, bilateral lung V_20_ ≤ 30%, V_30_ ≤ 20%, spinal cord D_max_ ≤ 45 Gy, heart V_30_ ≤ 40%, and V_40_ ≤ 30%. According to the tumor size and activity distribution, 3~4 high-dose peaks with a diameter of 1 cm and spacing of 3cm were arranged in a near-spherical distribution. This SFRT delivery was administered over 3 fractions,total dose 22.5Gy), immediately followed by conventionally fractionated radiotherapy to the residual solid tumor component 15 fractions,total dose 30Gy.

The dose selection was based on the need to overcome the radio−resistance of bulky tumors (peak dose 7.5Gy) while preserving surrounding normal tissue (valley dose 3Gy). For the sequential conventionally fractionated boost (15×2Gy), the biological effective dose (BED, α/β=10) was calculated as 36Gy, and the SFRT component contributed an additional 39.375Gy, resulting in a total BED of 75.375Gy for the re−irradiation course. When combined with the initial IMRT (57.5Gy/23 fractions, BED_10_ = 71.875Gy), the cumulative tumor BED_10_ reached 147.25Gy.

For OAR safety, a BED model with α/β=3 was applied. Considering the 17−month interval between treatments, a conservative 50% residual contribution from the first course was assumed. The cumulative BED_3_ values were: spinal cord ≈ 35.90Gy, total lung mean ≈ 18.26Gy, heart mean ≈ 21.79Gy, and right lung mean ≈ 28.80Gy. All these values remain within commonly accepted re−irradiation constraints (spinal cord BED_3_ <50Gy, lung mean BED_3_<40Gy, heart mean BED_3_<45Gy), confirming that the intensified regimen did not compromise OAR tolerance.

The dose for 95% of the PTV was 3000cGy in 15 fractions, with GTV-H V_50_ = 100% followed by ROI. The D_max_, D_98_, D_95_, D_50_, and D_2_ doses were 6026.57 cGy, 5550.67 cGy, 5589.32 cGy, 5765.74 cGy, and 5937.34 cGy, respectively, in accordance with the prescription requirements. The OAR doses were as follows: right lung (Lung-R) D_mean_ = 861.74 cGy, V_20_ = 14.02%, V_30_ = 3.28%; left lung (Lung-L) D_mean_ = 375.88 cGy, V_20_ = 0%, V_30_ = 0%; spinal cord D_max_ = 946.69 cGy; and Heart D_mean_ =949.6 cGy, V_30_ = 0.8%, V_40_ = 0%. SFRT effectively controlled the dose to surrounding normal tissues within tolerable limits, and the lung metastasis also achieved PR after 288 days (See **Figure 2**).

## Discussion

The use of minimally invasive ablative techniques is becoming increasingly vital for treating and managing colorectal cancer and associated liver and lung metastases. Stereotactic body radiotherapy (SBRT) is a cornerstone modality, with systematic evidence supporting its efficacy, safety and precision achieved through advanced imaging and dosimetric planning. Concurrently, CT-guided high-dose-rate brachytherapy (CT-BRT) has emerged as an effective metastasis-directed therapy, even in heavily pretreated patients, demonstrating high rates of disease control, minimal toxicity and potential for improved survival. Dosimetrically, CT-BRT offers favorable target coverage and reduced organ-at-risk exposure compared to SBRT, establishing it as a superior alternative in selected cases. The evolving landscape, including techniques like electron beam therapy, underscores the need to individualize modality selection based on tumor anatomy and prior treatments to optimize outcomes. Together, these modalities provide a versatile and potent arsenal for oligometastatic CRC (16, 23, 43, 55, 57–59). Advanced colon cancer patients often have poor physical conditions after multiple lines of chemotherapy, making them unable to tolerate further antitumor therapies. Radiotherapy is generally used palliatively for metastases, and there is limited evidence regarding its application to primary colon lesions, especially in combination treatment approaches (58, 60, 61). Consequently, patients with poor performance status who cannot tolerate surgery or further systemic chemotherapy have limited treatment options highlighting the critical role of advanced radiotherapy modalities in this patient population. In this clinical dilemma, the RISE trial (21) demonstrates that adding radiotherapy when systemic therapy proves insufficient can reduce the risk of thoracic tumor progression, thereby providing evidence for management strategies in such patients (21, 22). A case of advanced colon cancer with severe local intestinal obstruction, cough and hemoptysis is reported, in which oART was successfully delivered to the primary colon lesion and SFRT was administered to a large lung metastasis. No radiotherapy-related toxicities, such as radiation proctitis, intestinal perforation, radiation pneumonitis, or bone marrow suppression, were observed during or after treatment. Follow-up chest and abdominal CT scans at days 26, 75, 120, 200, 230, 263, and 330. **Table 1** documents the treatment course for the FBCT-guided oART. According to the RECIST 1.1 criteria, this intestinal lesion is classified as a non-measurable lesion, and its treatment response should be comprehensively evaluated alongside clinical symptoms. The overall trend demonstrates continuous improvement, with the largest diameter significantly reduced from 43.79 mm at baseline to 16.10 mm at the endpoint, an overall change rate of -63.23%. It is noteworthy that although fluctuations in lesion size were observed during treatment, the overall trend maintained reduction. Corresponding with radiographic findings, the patient’s clinical symptoms showed remarkable improvement, with the abdominal pain score decreasing from 5/10 at baseline to 0/10, bowel obstruction was completely resolved, treatment-related toxicities were mild (all ≤ Grade 1), and the ECOG score improved from 3 and remained at 1, adequately demonstrating treatment efficacy.

The administration of online ART in advanced colon cancer are usually faced with multiple challenges, contributing to of the limited clinical case reports. Intven et al. (62) reported the use of oART for rectal cancer treatment, with a total dose of 25 Gy and an average treatment time of 48 minutes. De Jong et al (63) found that most rectal cancer patients required approximately 34 minutes of treatment time. In contrast, the average treatment time in this case was 32 minutes, indicating that this approach not only improved treatment precision and safety but also enhanced efficiency while ensuring patient tolerance. Although most oART is currently implemented on MRI-based platforms (64) due to superior soft tissue visualization and reduced radiation exposure, IMRT typically relies on CBCT and FBCT for assessing target volume changes (65, 66). However, these imaging modalities have inherent limitations, such as low contrast and unclear tumor – normal tissue boundaries, which may compromise treatment precision, increase radiation exposure to normal tissues and increase the risk of radiotherapy-related toxicities. On the contrary, FBCT-guided online ART offers cost effective, rapid image acquisition, which aligns well with the requirements of adaptive radiotherapy (57, 67, 68). Additionally, FBCT has high system integration and broad applicability, making it more convenient for future widespread adoption (33, 69). Following FBCT-guided online ART, the patient in this case demonstrated a reduction rate exceeding 60% in intestinal lesions during the 11-month follow-up period. This favorable treatment response indicates the efficacy of this technical approach (59). Consequently, the findings of this study suggest that for medical centers not yet equipped with MRI-guided radiotherapy systems, FBCT-guided online adaptive radiotherapy may serve as a practical and cost-effective alternative.

SFRT has shown great potential in treating large-volume tumors that are resistant to conventional therapies (70, 71). SFRT demonstrates high clinical response rates and minimal toxicity in large-volume primary or metastatic malignancies by achieving highly heterogeneous dose distribution in three-dimensional space. This technology may be particularly suitable for large-volume tumors (diameter > 5 cm) and radio-resistant solid tumors, including advanced tumor with poor performance status. Research indicates that SFRT can induce potent distant effects and immunogenic cell death. Therefore, the combination of SFRT with immune checkpoint inhibitors (ICIs) may enhance treatment outcomes in metastatic cancers through synergistic therapeutic effects. As summarized in **Table 2**, the patient received SFRT for the lung tumor 26 days after initiating intestinal radiotherapy. This treatment regimen exhibited sustained therapeutic effects, with the largest tumor diameter significantly decreasing from 53.19 mm at baseline to 21.16 mm at Day 266 (overall change rate of -60.26%). While mild enlargement was observed during later treatment phases, the patient’s clinical symptoms maintained a steadily improved trend throughout the treatment course, with significant relief in cough and hemoptysis. The ECOG score improved from 3 at baseline to 1 and remained stable, reflecting the positive impact of treatment on quality of life. Future studies explore not only how to optimize combination with other therapies such as targeted therapy and immunotherapy, but also combination with other forms of radiotherapy (71–73).

The limitations of this study include the need for better soft tissue resolution, improved target re-contouring, reduced plan evaluation time, increased speed, optimized staff allocation and a longer follow-up period. Another key focus of this study is optimizing microbeam array configurations for SFRT (such as peak-to-valley dose ratio, spacing and number of peak regions) while innovating methods for normal tissue protection to maximize therapeutic benefits within safe parameters. This case, however, proves the safety and efficacy of using uRT-Linac506c KV-FBCT-guided online ART combined with SFRT in the treatment of advanced colon cancer with emergencies. The strategy has markedly improved the patient’s clinical symptoms and achieved significantly local response. It provides a new treatment option for patients with advanced colon cancer.

## Conclusion

This case details the first-time use of FBCT-guided oART for a primary ascending colon tumor and subsequent SFRT re-irradiation for a bulky pulmonary oligometastasis. Surprisingly, this strategy led to precise control of both lesions and symptom relief, with no related adverse events observed. At 11- month post-treatment review, the therapeutic response remained at PR, including continuous improvement of performance status. This approach offers a safe and effective individualized radiotherapy paradigm for advanced refractory colorectal cancer. Importantly, this approach provides a practical clinical pathway for institutions without MRI-linac systems to deliver precision adaptive radiotherapy and re-irradiation, thereby broadening access to such advanced technologies for advanced cancer patients. However, further preclinical studies are necessary to explore the therapeutic efficacy of combining radiotherapy with immunotherapy for colorectal cancer with lung metastases.

### Statistics

The adaptive radiotherapy plan (ARTplan1) was mapped onto subsequent CT images via deformable image registration. Dose distribution was calculated using a dedicated algorithm to generate a virtual non-adaptive radiotherapy plan (IGRTplan) as the control group. Statistical analyses of dose parameters for target volumes (TV) and organs at risk (OAR) were performed using SPSS 26.0. The Mann-Whitney U test was employed for non-normally distributed data. Dose-volume histograms and blood count results were refined into line charts using Origin version 64.

## Data Availability

The original contributions presented in the study are included in the article/supplementary material. Further inquiries can be directed to the corresponding authors.
